# Renal-Protective Effects and Potential Mechanisms of Traditional Chinese Medicine after Ischemia-Reperfusion Injury

**DOI:** 10.1155/2021/5579327

**Published:** 2021-02-19

**Authors:** Demin Liu, Songling Tang, Lu Gan, Wei Cui

**Affiliations:** ^1^Cardiology Department, Second Hospital of Hebei Medical University, Shijiazhuang, Hebei 050000, China; ^2^Research Laboratory of Emergency Medicine, Department of Emergency Medicine, National Clinical Research Center for Geriatrics, West China Hospital, Sichuan University, Chengdu 610041, China

## Abstract

Renal ischemia-reperfusion (I/R) injury mainly causes acute kidney injury (AKI) after renal transplantation, trauma, sepsis, and hypovolemic shock. Patients with renal I/R injury are frequently associated with a poor prognosis. Traditional Chinese medicine (TCM) has been used for the prevention and treatment of various diseases in China and other Asian countries for centuries. Many studies have shown the protective effect of TCM on renal I/R injury, due to its diverse bioactive components. The potential mechanisms of TCMs on renal I/R injury include anti-inflammation, antioxidative effect, anti-cell death, downregulation of adhesion molecule expression, regulation of energy metabolism by restoring Na^+^-K^+^-ATPase activity, and mitochondrial fission. This review summarizes the major developments in the effects and underlying mechanisms of TCMs on the renal I/R injury.

## 1. Introduction

Renal ischemia-reperfusion (I/R) injury is the main cause of acute kidney injury (AKI) and is reported to be associated with high morbidity and mortality in both adults and children. It often occurs after renal transplantation, as well as in sepsis, trauma, hypovolemic shock, etc. [1]. During the renal I/R process, excess reactive oxygen species (ROS) are likely to cause oxidative stress, which subsequently triggers lipid peroxidation [2] and cell death due to apoptosis, autophagy, pyroptosis, and ferroptosis caused by DNA and protein damage in ischemic tissue [3]. In addition, inflammation plays an important role in renal I/R injury, along with leukocyte infiltration and tissue damage in the kidney, followed by excessive production of proinflammatory cytokines [4]. Energy metabolism dysfunction, such as decreased Na^+^-K^+^-ATPase activity, has been considered as one of the critical mechanisms of renal I/R injury [5].

Increase in blood urea nitrogen (BUN) and creatinine (Cr) [3] and a higher urinary total protein level are reported to occur after renal I/R injury [6]. Histopathology of renal I/R injury shows luminal narrowing, intraluminal necrotic cellular debris, tubular basal membrane rupture, tubular vacuolization, interstitial congestion, apparent cell swelling and nuclear infiltration in the H&E staining of renal tissues [7], and higher Kim-1 expression, thus causing a higher renal tubular injury score [2].

Traditional Chinese medicine (TCM) has been used in China, Korea, Japan, and other Asian countries for the treatment of various diseases, such as cerebrovascular diseases, cardiac diseases, liver diseases, renal disorders, etc. In the theory of TCM, renal I/R injury is attributed to Qi and blood deficiency and blood stasis syndrome [8]. From the perspective of TCM, renal I/R injury should be treated through the replenishment of Qi and activation of blood circulation [7]. Several TCMs have been applied for the prevention and treatment of renal I/R, and they show different renal-protective effects in Table 1, including anti-inflammation (*Cordyceps sinensis*, cordycepin, Yisheng injection, cryptotanshinone, *Panax notoginseng*, total glucosides of paeony, hydroxysafflor yellow A, farnesiferol B, anemoside B4, etc.), antioxidative stress (ligustrazine, berberine, glycyrrhizin, Sheng Mai San, hyperoside, salvianolic acid A, salvianolic acid B, arctigenin, farnesiferol B, polydatin, Shenfu injection, etc.), anti-cell death (honokiol, cryptotanshinone, brazilin, salvianolic acid B, Sanqi oral solution, etc.), downregulation of adhesion molecule expression (ligustrazine and Yisheng injection), regulation of energy metabolism by restoring Na^+^-K^+^-ATPase activity (Fufang Shenhua Tablet), and mitochondrial fission (hyperoside).

In this review, we have discussed the studies in the past decades on the protective effects and potential mechanisms of TCMs and the major ingredients on renal I/R injury.

## 2. Protective Effects of TCMs and Major Ingredients on Renal I/R Injury

Generally speaking, almost all the following TCMs and the major ingredients play a protective role in renal I/R injury, including the improvement of renal function and histological changes in renal tissues, inhibiting inflammatory cytokine release and macrophage/neutrophil infiltration, decreasing production of oxidative stress and lipid oxidation, regulating programmed cell death (apoptosis, autophagy, pyroptosis, or ferroptosis), decreasing release of adhesion molecules, regulating energy metabolism, endothelial injury, and mitochondrial dysfunction [25].

### 2.1. Renal Function and Renal Histological Examination

In previous studies, renal function was often measured by the levels of Scr and BUN. In some cases, urinary total protein level was also used for renal function measurement. It was reported that Scr and BUN levels were significantly increased in the I/R model group, compared with the sham group. TCMs or CCMs, such as Fufang Shenhua Tablet, cryptotanshinone, and notoginsenoside R1, could inhibit the increased levels of Scr and BUN, and thus improve renal function [13, 17, 19]. In addition, anemoside B4 could decrease the urinary total protein level [6], indicating improvement of renal function.

Several histological abnormalities were observed after renal I/R injury, including tubular brush border loss, epithelial cell dilatation and necrosis, cytoplasmic vacuolization, and cast formation [3]. Most TCMs were reported to alleviate renal histological changes and decrease renal tubular injury score, caused by I/R injury. Furthermore, TCMs, such as Sanqi oral solution, hydroxysafflor yellow A, and total glucosides of paeony, showed dose-dependent effects, such that higher concentrations showed greater alleviating effects [4, 7, 23]. In contrast, ATG played a detrimental role in renal I/R injury since renal function and histology worsened with increasing concentration [3].

### 2.2. Anti-Inflammatory Activity

Most TCMs play an anti-inflammatory role in renal protection after I/R injury. Increase in expressions of inflammatory genes, such as MCP-1 and TNF-a mRNA, and their proteins were observed in the renal I/R group. *Cordyceps sinensis* treatment was reported to reverse these effects [9, 26]. Additionally, cordycepin, the extract from *Cordyceps sinensis*, could decrease secretion of proinflammatory factors and alleviate inflammatory reaction [10], including IL-1*β*, IL-6, and TNF-*α*, at the protein and mRNA levels. The same effect was observed after treatment with Yisheng injection, Fufang Shenhua Tablet, glycyrrhizin, honokiol, cryptotanshinone, notoginsenoside R1, brazilin, total glucosides of paeony, hydroxysafflor yellow A, arctigenin, farnesiferol B, and anemoside B4 [2–4, 6, 8, 12, 13, 15–17, 19, 20, 23]. Moreover, the infiltration of neutrophils and macrophages was observed in some studies. ATG decreased the infiltration of CD11b + Gr1+ neutrophils and CD68+ macrophages in renal tissue after I/R injury [3], and the neutrophil infiltration was decreased after pretreatment with Yisheng injection [12].

### 2.3. Antioxidative Stress

Oxidative stress and lipid oxidation increase after renal I/R injury, while treatment with some TCMs inhibits the production of oxidative stress and promotes the production of antioxidant factors. In Feng Han and AR Shahed's studies [10, 26], *Cordyceps sinensis* and its extract cordycepin increased the levels of antioxidative factors, including GSH, GSHPx, and SOD, and decreased the levels of oxidative stress products, including MDA, NO, and iNOS. Other TCMs also showed similar effects, such as MDA decrease and SOD increase in the ligustrazine treatment group [11], berberine treatment group, and Sal A and Sal B treatment groups [21, 22], SOA decrease and SOD increase after glycyrrhizin treatment [15], MDA/MPO decrease and SOD/CAT increase after honokiol treatment [16], decrease in iNOS and ONOO^–^levels after SMS treatment [27], and decrease in ROS levels after hyperoside treatment. CS treatment decreased NGAL levels both in kidney tissues and in urine, which was increased in the I/R group [28]. In addition, farnesiferol B reduced oxidative stress (diminished levels of NAGL and H2O2) and lipid oxidative signaling pathways (diminished levels of lipid peroxidation markers, including 4-HNE and MDA) in renal I/R [2].

### 2.4. Anti-Cell Death

There are several types of programmed cell death, including apoptosis, autophagy, pyroptosis, and ferroptosis. Previous studies have reported that the protective effect of TCMs on renal I/R injury was associated with programmed cell death, especially apoptosis.

Antiapoptosis was observed after treatment with some TCMs before or after renal I/R injury, with downregulation of the levels of apoptosis proteins and upregulation of the levels of antiapoptosis proteins. In the *Cordyceps sinensis* treatment group [26], Fas and FasL mRNA expression levels were decreased, while caspase-3 activity was decreased after glycyrrhizin and honokiol treatment [15, 16]. TUNEL-positive cells were increased after Sal A treatment, indicating a reduction of apoptotic activity [21]. An *in vitro* study found that berberine significantly inhibited the expression of apoptotic proteins, such as Bax [14].

Treatment with Sanqi (SQ) oral solution showed decrease in apoptosis and increase in autophagy [7]. Lower Bax and caspase-3 levels and higher Bcl-2 level were observed in the SQ treatment group in a dose-dependent manner, demonstrating the antiapoptotic activity of SQ. Meanwhile, compared to the I/R model group, higher dose of SQ significantly increased the levels of LC3II/LC3I and Beclin1, indicating an enhanced autophagy activity after renal I/R injury. In addition, treatment with SQ and 3-MA, an autophagy inhibitor, showed worse histological damages and renal function, compared with SQ alone treatment, indicating the essential role of autophagy in SQ treatment during renal I/R injury.

In Yu Pang's study [22], the levels of GSDMD, caspase-1, and IL-1*β*, the pyroptosis-related proteins, were strongly upregulated in the model group. The pyroptosis-related proteins were significantly decreased after treatment with Sal B, demonstrating the antipyroptosis activity of Sal B.

Lastly, ferroptosis plays an important role in the protective effects of farnesiferol B treatment on renal I/R injury. The mRNA expression of Gpx4, the key ferroptosis regulator, was significantly reduced after renal I/R injury, while farnesiferol B treatment increased its expression. However, the intergroup difference was not significant [2]. So, further studies are needed.

### 2.5. Downregulation of Adhesion Molecules

In the I/R model group, expression of ICAM-1 was upregulated but was greatly diminished after ligustrazine treatment [11]. Moreover, ICAM-1 was downregulated in a dose-dependent manner after pretreatment with Yisheng injection in the renal I/R group [12].

### 2.6. Recovery of Na^+^-K^+^-ATPase Activity

Yang Yang et al. [5] found a significant decrease in Na^+^-K^+^-ATPase activity in the I/R model group when compared with the sham group. However, Shenhua Tablet (SHT) was observed to increase the decreased Na^+^-K^+^-ATPase activity after renal I/R, both in the low-dose group and the high-dose group. Combined with other results in this study, the authors concluded that SHT could recover the number of Na^+^-K^+^-ATPase and improve its activity in the tubular epithelial cells, promote epithelial cell polarity in renal tubular cells, and thereby improve renal function.

### 2.7. Endothelial Protective Effects

Salvianolic acid A (SAA) can protect peritubular capillary endothelium from renal I/R injury [29]. VEGFA expression was increased in tubular epithelial cells, which was associated with peritubular capillary density in the SAA treatment group, as well as the reduced levels of plasma vWF and lower platelet activation after I/R injury. Moreover, significant increase in Klotho protein expression was observed, indicating less endothelial injury after renal I/R with SAA treatment.

## 3. Potential Mechanism of TCMs and Major Ingredients in Renal I/R Injury

Several signaling pathways were considered to be involved in the renoprotective effects of TCMs, especially the anti-inflammation effect, antioxidative effect, antiapoptotic activity, and some others. These pathways included the NF-*κ*B/TLR4, NF-*κ*B-p65, NF-*κ*B-p MAPK, Nrf2-NLRP3, Nrf2-HO-1, Shh signaling pathway, PI3K-Akt [30], ERK/mTOR [7], Akt/mTOR/4EBP1 [21], OMA1-OPA1 [18], XIST/MicroRNA-124-3p/ITGB1 [23], etc. The most important pathways involved in renal I/R injury are discussed below.

### 3.1. NF-*κ*B Signaling Pathway

NF-*κ*B is believed to play an important role in the anti-inflammatory effects and antiapoptotic and antioxidative activity of TCMs in renal I/R process.

Activation of the NF-*κ*B signaling pathway was inhibited in the cryptotanshinone (CTS) treatment group after renal I/R [17]. I/R significantly increased the phosphorylated p65 and I*κ*B*α* levels, indicating the activation of NF-*κ*B signaling pathway. After CTS treatment, the phosphorylation of p65 and I*κ*B*α* was inhibited. Brazilin was also proven to downregulate the inflammatory activity by inhibiting NF-*κ*B signaling pathway activation *in vitro* [20].

Hydroxysafflor yellow A (HSYA) was reported to decrease the renal inflammation after I/R injury by suppressing the TLR4/NF-*κ*B signaling pathway activation [4], as phospho-IKK*β*, phospho-I*κ*B*α*, and NF-*κ*B-p65 expression levels were upregulated in the I/R group but were downregulated after treatment with HSYA *in vitro*. Similar effect of arctigenin (ATG) and polydatin on TLR4 and NF-*κ*B-p65 expression was observed [3, 31].

Notoginsenoside R1 (NR1) decreased inflammatory factors and apoptosis by inhibiting the phosphorylation of p38 MAPK and NF-*κ*B activation in renal tissue after I/R injury [12]. The anti-inflammatory effects and antioxidative activities of farnesiferol B on renal I/R injury were based on the activation of TGR5 and inhibition of the NF-*κ*B pathway [2].

### 3.2. Nrf2 Signaling Pathway

In renal I/R process, Nrf2 plays a central role in renal-protective effects of TCMs, including the anti-inflammatory effects and antioxidative and anti-cell death activity.

Cordycepin is known to alleviate inflammation, oxidative stress, and apoptosis in the I/R process. The antioxidative mechanism of cordycepin was analyzed by measuring the levels of Nrf2 and HO-1, the key proteins in antioxidative stress process. Western blotting results showed an increase in the expression of Nrf2 and HO-1 after cordycepin treatment, indicating that cordycepin may suppress the oxidative stress during renal I/R injury through the Nrf2/HO-1 signaling pathway [10].

Another study found that Sal B could inhibit caspase-1/GSDMD-mediated pyroptosis and thereby alleviate I/R injury in mice, by activating Nrf2/NLRP3 signaling pathway [22]. Sal B was reported to inhibit pyroptosis by downregulating pyroptosis-related proteins GSDMD, caspase-1, and IL-1*β*. Moreover, Sal B showed its antioxidative property by inhibiting the expression of Keap1 protein, thus increasing Nrf2 nuclear expression and HO-1 protein levels *in vivo*. *In vitro*, Sal B reduced NLRP3 protein expression and increased Nrf2 nuclear import and activated expression of downstream antioxidant components. Moreover, the Nrf2/NLRP3 signaling pathway was demonstrated to be involved through Nrf2 knockdown *in vivo* and siNrf2 transfection *in vitro*.

### 3.3. Sonic Hedgehog (Shh) Signaling Pathway

Shh signaling pathway is important in the antioxidative and antiapoptotic effects of TCM treatment after renal I/R injury. Polydatin alleviated apoptosis and oxidative stress after renal I/R injury through activating the sonic hedgehog signaling pathway [24]. Polydatin was found to dose-dependently induce increase in the Shh gene and protein expression compared with the I/R group, both *in vitro* and *in vivo*. Moreover, the mRNA and protein expression of Ptch 1 and Smo, two key elements in the Shh signaling pathway, were reduced by cyclopamine (an inhibitor of Smo) combined with polydatin. Meanwhile, higher apoptotic activity and oxidative effects were observed. Therefore, inhibition of Shh signaling impaired the antioxidative and antiapoptotic effects of polydatin.

## 4. Summary

This review discusses the protective effects of TCMs and their active components on renal I/R injury. The mechanisms of the protective effects of TCMs include inhibition of inflammation, decrease of oxidative stress and lipid oxidation products, regulation of programmed cell death, reduction of adhesion molecule release, and regulation of energy metabolism and endothelial injury by activating NF-*κ*B and Nrf2 signaling pathways, which are considered to be multiple targets for successful treatment. However, the existing studies are either animal experiments *in vivo* or *in vitro* studies, with lower level of evidence and strength of recommendation. Therefore, further clinical studies are needed to explore the effects of TCMs and the underlying mechanisms.

## Figures and Tables

**Table 1:** The protective effects and potential mechanisms of TCM and the major ingredients on renal I/R injury.

TCM or its ingredients	Experiment object	Protective effects	Potential mechanisms	Ref.
*Cordyceps sinensis*	SD rats	Decreased inflammation	—	[9]
Cordycepin	SD rats	Decreased inflammation, apoptosis, oxidative stress	Nrf2-HO-1	[10]
Ligustrazine	C57BL/6 mice	Decreased apoptosis, oxidative stress	—	[11]
Yisheng injection	C57BL/6 mice	Reduced neutrophil infiltration, decreased inflammation	—	[12]
Fufang Shenhua Tablet	Wistar rats	Decreased inflammation	Downregulation of TLRs	[8]
	Wistar rats	Decreased inflammation	MyD88 signaling pathway	[13]
	Wistar rats	Decreased Na + -K + -ATPase level	—	[5]
Berberine	NRK-52E cells	Decreased mitochondrial oxidative stress and apoptosis	Sirt1/p53 signaling pathway	[14]
Glycyrrhizin	C57BL/6 mice	Inhibition of inflammation and renal cell apoptosis	p38 MAPK signaling pathway	[15]
Honokiol	Wistar albino rats	Inhibition of oxidative stress and inflammation	—	[16]
Cryptotanshinone	C57BL/6 mice	Inhibition of cell apoptosis and inflammatory response	NF-*κ*B-p38 MAPK signaling pathway	[17]
Hyperoside	C57BL/6 mice	Attenuated tubular cell apoptosis, oxidative stress, inhibited mitochondrial fission	OMA1-OPA1 axis	[18]
	HK-2 cells			
Notoginsenoside R1	SD rats	Reduced apoptosis and inflammatory response	NF-*κ*B-p38 MAPK signaling pathway	[19]
Brazilin	SD rats	Decreased inflammation attenuated apoptosis and oxidative stress	NF-*κ*B signaling pathway	[20]
Salvianolic acid A	SD rats		Akt/mTOR/4EBP1 pathway	[21]
	HK-2 cells			
Salvianolic acid B	BALB/c mice	Reduced oxidative stress and inflammation, inhibition of pyroptosis	Nrf2/NLRP3 signaling pathway	[22]
Total glucosides of paeony	SD rats	Attenuated apoptosis and inflammation	XIST/miR-124-3p/ITGB1 axis	[23]
	NRK-52E cells			
Hydroxysafflor yellow A	SD rats	Decreased inflammation	TLR4/NF-*κ*B pathway	[4]
Sanqi oral solution	SD rats	Enhanced autophagy, attenuated apoptosis	ERK/mTOR pathway	[7]
Arctigenin	C57BL/6 mice	Alleviated inflammatory response and oxidative stress	NF-*κ*B signaling pathway	[3]
Farnesiferol B	Female C57/BJ mice	Attenuated inflammation	TGR5/NF-*κ*B signaling pathway	[2]
Polydatin	BALB/c mice	Antiapoptosis and antioxidative effects	Sonic hedgehog (shh) signaling pathway	[24]
	Primary renal tubular epithelial cells			

## References

[1] Sun Z., Wang X. (2020). Protective effects of polydatin on multiple organ ischemia-reperfusion injury. *Bioorganic Chemistry*.

[2] Zhang L., Fu X., Gui T. (2019). Effects of Farnesiferol B on ischemia-reperfusion-induced renal damage, inflammation, and NF-*κ*B signaling. *International Journal of Molecular Sciences*.

[3] Han F., Xia X.-X., Dou M. (2018). Arctigenin: a two-edged sword in ischemia/reperfusion induced acute kidney injury. *Biomedicine & Pharmacotherapy*.

[4] Bai J., Zhao J., Cui D. (2018). Protective effect of hydroxysafflor yellow A against acute kidney injury via the TLR4/NF-*κ*B signaling pathway. *Scientific Reports*.

[5] Yang Y., Wei R.-B., Zheng X.-Y. (2014). Effects of compound Shenhua tablet on renal tubular Na+-K+-ATPase in rats with acute ischemic reperfusion injury. *Chinese Journal of Integrative Medicine*.

[6] Li J., Zuo S. S., Qiu X. X. (2020). [Study on therapeutic effect and its related mechanism of anemoside B4 on ischemia reperfusion injury induced by renal artery and vein ligation in rats]. *Zhongguo Zhong Yao Za Zhi*.

[7] Tian R., Wang P., Huang L. (2020). Sanqi oral solution ameliorates renal ischemia/reperfusion injury via reducing apoptosis and enhancing autophagy: involvement of ERK/mTOR pathways. *Frontiers in Pharmacology*.

[8] Zheng X.-Y., Wei R.-B., Shi S.-Z., Yin Z., Chen X.-M. (2012). Effects of Fufang Shenhua tablet on the expression of toll-like receptors during acute kidney injury induced by ischemia-reperfusion in rats. *Chinese Journal of Integrative Medicine*.

[9] Wang H.-P., Liu C.-W., Chang H.-W., Tsai J.-W., Sung Y.-Z., Chang L.-C. (2013). Cordyceps sinensis protects against renal ischemia/reperfusion injury in rats. *Molecular Biology Reports*.

[10] Han F., Dou M., Wang Y. (2020). Cordycepin protects renal ischemia/reperfusion injury through regulating inflammation, apoptosis, and oxidative stress. *Acta Biochimica et Biophysica Sinica*.

[11] Feng L., Xiong Y., Cheng F., Zhang L., Li S., Li Y. (2004). Effect of ligustrazine on ischemia-reperfusion injury in murine kidney. *Transplantation Proceedings*.

[12] Feng L., Guo Y., Cheng F. (2010). Effects of pretreatment with Yisheng injection on renal warm ischemia/reperfusion injury in mice. *Transplantation Proceedings*.

[13] Li Q.-P., Wei R.-B., Yang X. (2019). Protective effects and mechanisms of Shenhua tablet (肾华片) on toll-like receptors in rat model of renal ischemia-reperfusion injury. *Chinese Journal of Integrative Medicine*.

[14] Lin Y., Sheng M., Ding Y. (2018). Berberine protects renal tubular cells against hypoxia/reoxygenation injury via the Sirt1/p53 pathway. *Journal of Natural Medicines*.

[15] Ye S., Zhu Y., Ming Y., She X., Liu H., Ye Q. (2014). Glycyrrhizin protects mice against renal ischemia-reperfusion injury through inhibition of apoptosis and inflammation by downregulating p38 mitogen-activated protein kinase signaling. *Experimental and Therapeutic Medicine*.

[16] Yu Y., Li M., Su N. (2016). Honokiol protects against renal ischemia/reperfusion injury via the suppression of oxidative stress, iNOS, inflammation and STAT3 in rats. *Molecular Medicine Reports*.

[17] Bai T., Yang K., Qin C., Xu T., Yu X., Zhang J. (2019). Cryptotanshinone ameliorates renal ischaemia-reperfusion injury by inhibiting apoptosis and inflammatory response. *Basic & Clinical Pharmacology & Toxicology*.

[18] Wu L., Li Q., Liu S. (2019). Protective effect of hyperoside against renal ischemia-reperfusion injury via modulating mitochondrial fission, oxidative stress, and apoptosis. *Free Radical Research*.

[19] Liu W.-J., Tang H.-T., Jia Y.-T. (2010). Notoginsenoside R1 attenuates renal ischemia-reperfusion injury in rats. *Shock*.

[20] Jia Y., Zhao J., Liu M. (2016). Brazilin exerts protective effects against renal ischemia-reperfusion injury by inhibiting the NF-*κ*B signaling pathway. *International Journal of Molecular Medicine*.

[21] Song Y., Liu W., Ding Y. (2018). Salvianolic acid A ameliorates renal ischemia/reperfusion injury by activating Akt/mTOR/4EBP1 signaling pathway. *American Journal of Physiology-Renal Physiology*.

[22] Pang Y., Zhang P. C., Lu R. R. (2020). Andrade-oliveira salvianolic acid B modulates caspase-1-mediated pyroptosis in renal ischemia-reperfusion injury via Nrf2 pathway. *Frontiers in Pharmacology*.

[23] Chen F., Hu Y., Xie Y. (2020). Total glucosides of paeony alleviate cell apoptosis and inflammation by targeting the long noncoding RNA XIST/MicroRNA-124-3p/ITGB1 axis in renal ischemia/reperfusion injury. *Mediators of Inflammation*.

[24] Meng Q.-H., Liu H.-B., Wang J.-B. (2016). Polydatin ameliorates renal ischemia/reperfusion injury by decreasing apoptosis and oxidative stress through activating sonic hedgehog signaling pathway. *Food and Chemical Toxicology*.

[25] Zeng Z., Chen Z., Xu S. (2016). Polydatin protecting kidneys against hemorrhagic shock-induced mitochondrial dysfunction via SIRT1 activation and p53 deacetylation. *Oxidative Medicine and Cellular Longevity*.

[26] Shahed A. R., Kim S. I., Shoskes D. A. (2001). Down-regulation of apoptotic and inflammatory genes by Cordyceps sinensis extract in rat kidney following ischemia/reperfusion. *Transplantation Proceedings*.

[27] Lee I., Lee C., Chang C., Chien C., Lin M. (2005). Sheng mai san, a Chinese herbal medicine, protects against renal ischaemic injury during heat stroke in the rat. *Clinical and Experimental Pharmacology and Physiology*.

[28] Yu H., Zhou Q., Huang R., Yuan M., Ao X., Yang J. (2012). [Effect of cordyceps sinensis on the expression of HIF-1*α* and NGAL in rats with renal ischemia-reperfusion injury]. *Zhong Nan Da Xue Xue Bao Yi Xue Ban*.

[29] Zhang Z., Qi D., Wang X. (2018). Protective effect of Salvianolic acid A on ischaemia-reperfusion acute kidney injury in rats through protecting against peritubular capillary endothelium damages. *Phytotherapy Research*.

[30] Liu H. B., Meng Q. H., Huang C., Wang J. B., Liu X. W. (2015). Nephroprotective effects of polydatin against ischemia/reperfusion injury: a role for the PI3K/Akt signal pathway. *Oxidative Medicine and Cellular Longevity*.

[31] Li Y., Xiong W. J., Yang J. (2014). [Inhibitory effect of polydatin on expression of toll-like receptor 4 in ischemia-reperfusion injured NRK-52E cells]. *Zhongguo Zhong Yao Za Zhi*.

